# Cost-Sensitive Learning for Emotion Robust Speaker Recognition

**DOI:** 10.1155/2014/628516

**Published:** 2014-06-04

**Authors:** Dongdong Li, Yingchun Yang, Weihui Dai

**Affiliations:** ^1^School of Information Science and Engineering, East China University of Science and Technology, Shanghai 200237, China; ^2^Department of Computer Science and Technology, Zhejiang University, No. 38, Yuquan Road, Zhejiang 310027, China; ^3^School of Management, Fudan University, No. 220, Handan Road, Shanghai 200433, China

## Abstract

In the field of information security, voice is one of the most important parts in biometrics. Especially, with the development of voice communication through the Internet or telephone system, huge voice data resources are accessed. In speaker recognition, voiceprint can be applied as the unique password for the user to prove his/her identity. However, speech with various emotions can cause an unacceptably high error rate and aggravate the performance of speaker recognition system. This paper deals with this problem by introducing a cost-sensitive learning technology to reweight the probability of test affective utterances in the pitch envelop level, which can enhance the robustness in emotion-dependent speaker recognition effectively. Based on that technology, a new architecture of recognition system as well as its components is proposed in this paper. The experiment conducted on the Mandarin Affective Speech Corpus shows that an improvement of 8% identification rate over the traditional speaker recognition is achieved.

## 1. Introduction

Biometric security systems are based on human exclusive and unique characteristics, such as fingerprints, face, voice, iris, and retina [1, 2]. These systems are used as an extra barrier to prevent unauthorized access to protect data by recognizing the users by their specific physiological or behavioral characteristic. This method is more reliable than the conventional method because it is based on “something one is” rather than “something one knows/has.”

With improved research of vocal signals, people's interactions through internet and mobile devices, such as phone banking, internet browsing, and secured information retrieval by voice, are becoming popular in a very rapid way [3]. There exists a need for greater security as these human-machine interactions over telephone lines and internet. At the same time, the powerful and ubiquitous handheld devices, such as smart phones and handheld computers, may contain a myriad of sensitive or personal information. All the applications mentioned above put great demand on speaker recognition based on modeling the speaker vocal tract characteristics, providing secure access to financial information (e.g., credit card information, bank account balance, etc.) or other sensitive customer information (e.g., healthcare records) [4]. Speaker verification provides an extra barrier to prevent unauthorized access to protect data and enhances the security offered by personal identification numbers or user selected passwords. It allows for contactless activation and mitigates the risks of stolen or lost keys, passwords, or keycards.

Automatic speaker recognition can be grouped into the following two classes: speaker verification and speaker identification. Speaker verification is the process to confirm the claim of identity and declare the person to be true or imposter. It is inclined to be used in security system using user specified passcodes for secure user logins. Speaker identification is the process to determine which one best matches the input voice sample from a pool of speakers' voices. Its main application area is forensics and investigation, where there is a need to determine the identifier of a person. According to the type of spoken utterances, speaker recognition can also be divided into three categories: text-independent, text-dependent, and text-prompted. In text-independent systems, an arbitrary phrase is uttered to recognize the speaker. In text-dependent systems, a fixed “voice password” is uttered. In “text-prompted” systems, an instruction is given to ask the speaker to utter a certain phrase.

Previous work on security-based speaker recognition systems largely falls within the domain of dealing with interspeaker variables, like channel and background noise. However, intraspeaker variables, like emotion and health state, can also cause an unacceptably high error rate, which limits the commercial viability of speaker recognition systems [5]. Driven by rapid ongoing advances in affective computing, speaker recognition with affective speech (SRAS) is now becoming a popular consideration of modern speaker recognition research. In real life, we cannot expect the speaker to be always in neutral or normal mood. Most of the speaker recognition systems enroll the speaker model with neutral speech. Such systems could distinguish speakers from the others accurately when the speaker provides neutral speech to identify. However, when the recognition step is faced with emotional speech, like angry speech or delighted speech, the systems suffer emotional state mismatch between training and testing stage and the performance deteriorates. We cannot afford to develop the speaker models in all possible emotions for improving the performance, which degrades the user-friendliness of the speaker recognition system. SRAS becomes important because of the difficulty in acquiring large number of affective speeches from the speakers.

In sophisticated human-machine interaction, equipping the computer with the affective computing ability so that it can recognize the identity of the user is urgently needed in many different applications [6]. In telecommunications, the telephone-based speech recognition performance can be enhanced with the SRAS systems. For example, in route emergency call applications which service for high priority emergency calls, the callers experience a panic and scary scene. Their voice is not neutral any more. In the meanwhile, SRAS can also facilitate the applications of call centre. In many cases, the speaker gets disappointed, anxious, or angry when they call to deal with after-sale services problems. SRAS can identify and assess the speaker and help the call centre quickly respond to the disputes as well as achieving the customers' satisfaction.

For such applications, it becomes necessary to take into account the affective impact of speaker recognition so that the speakers could be recognized even when there is only affective speech provided for testing. The focus of this work is to develop a robust intelligent human-machine interface, which is more adaptive and responsive to a speaker's identity in emotional environments. In this paper, we further the research work in [7] and apply cost-sensitive learning to optimize the classification procedure and system performance.

This paper is organized as follows. In the next section, we give a review to the related work. The emotional corpus and emotional speech analysis are introduced in Section 3.  Section 4 is committed to cost-sensitive learning and its application to speaker recognition.  Section 5 discusses the system architecture. The experiments comparison and result discussion are presented in Section 6. We close with a conclusion section.

## 2. Related Work

In the literature, there are a few studies that focus on speaker recognition with affective speech. Structure training [8, 9] is first proposed and noted as a promising approach to address this problem. The method attempts to elicit different manners of speaking during the enrollment and makes the system become familiar with the variation likely to be encountered in that person's voice. Emotion-added modeling method [10] also attempts to elicit different manners of speaking during the enrollment. The goal of the systems is to learn not only the true distribution of the speaker-dependent speech features but also the influences of various emotions that corrupt this distribution.

Most of such systems model the speakers with a variety of affective speech and achieve great success. Dongdong and Yingchun [11] construct the speaker models with clustered affective speech. This approach aims at the maximum utilization of the limited affective training speech data. The prosodic difference is exploited to cluster affective speech, and the corresponding models are built with the clustered speech for a given speaker.

Along the way, all these methods mentioned above ask users to provide additional reading (emotional) speech in the training stage, which would lead to the unfriendliness of the system.

On the contrary, other researchers aim to promote the SRAS performance with only neutral enrolled for training, by means of adding artificial affective information to neutral training speech or eliminating the affective information in the emotional testing speech. Feature domain compensation aims at adding emotional information to neutral acoustic features prior to model training. One example is the rules based feature modification method based on the statistics of prosodic features [12, 13]. Specifically, the rules of prosodic features modification of duration, pitch, and amplitude parameters are derived from a small number of the content matched source-target pairs. The speaker model is trained with an aggregation of data with all kinds of the converted affective speech and the neutral speech.

Krothapalli et al. [6] believe that performance of the speaker identification system developed using neutral features is better with transformed features compared to emotional features. He proposes neural network based feature transformation framework for mapping the time-aligned syllable level features from any specific emotion to neutral. Shahin investigates emotion identification when the database is biased towards different emotions based on each of HMMs [14] and SPHMMs [15, 16].

Besides, score domain compensation attempts to remove model score scales and shifts caused by varying affective conditions of speakers. An example of score domain compensation techniques is E-Norm [17]. By investigating the pitch distribution variation under different emotional states, Li et al. [7] propose an improved pitch envelope based frame-level score reweighted (PFLSR) algorithm to compensate the affective effect in both speaker verification and identification system. The PFLSR aims to separate the frames that have large speaker-emotion variability from the ones that are slightly affected by speakers' moods.

Most of the existing speaker recognition systems fail during affective speech due to emotional mismatch in the training and testing environments. Considering both the system friendliness and the algorithm complexity, a probability reweighted score domain compensation approach is proposed. The idea of score normalization has been long acknowledged to speaker verification at both utterance and frame level [18, 19]. It is widely used for its simplification, convenience, and excellent result. This work has furthered the study in [7] and used the supervised learning method to refine the final score.

## 3. Database and Speech Analysis

### 3.1. Database

The affective speech database evaluated in this paper is Mandarin Affective Speech Corpus (MASC) [20], which is distributed by the Linguistic Data Consortium. The speech in the database spans five different emotion types, which are neutral (unemotional), panic, anger, sadness, and elation. All the reading material is phonetically balanced, which covers all the phonemes in Chinese.

68 native speakers are elicited to utter 5 phrases and 20 sentences under five emotional states, as well as 2 extra neutral paragraphs speech. Each phrase and sentence is repeated for three times, respectively. Altogether the database contains 5,100 phrases (e.g., 5 phrases *3 times *68 subjects *5 emotional types), 20,400 utterances, and 136 paragraphs. The detailed material is described as follows.

- Five phrases: they are “shi de (yes),” “bu shi (no),” and three nouns as “ping guo (apple),” “huo che (train),” and “wang qiu (tennis ball).” In Chinese, these words contain many different basic vowels and consonants.
- 20 sentences: these sentences include 12 semantically neutral ones and 2 emotional ones for each type portraying the four emotional states. Different syntactical types are also represented in the sentences, which follow the material design of RUSLANA [21].
- Two paragraphs: they are two readings selected from a famous Chinese novel, stating a normal fact.

The MASC database is divided into three subsets: development set, training set, and evaluation set. The development set is composed of the speech from the first 18 people. The training set contains 50 speakers, whose 2 paragraphs of neutral speech are used to construct speaker models. The evaluation set is the utterance parts in five types of emotions. There are 50 such speakers, with 15000 authentic tests and 735000 imposter tests.

### 3.2. Speech Analysis

Referring to the affective speech, the prosody is a medium of emotional expression [22]. Phonetic research demonstrates that prosodic information involves complex articulatory and cognitive controls in speech [23–25]. Promising results of emotion classification have been achieved with the analysis of prosodic feature [26–28].

Prosody feature refers to the variables (range, contour, and jitter) of pitch, speaking rate, and intensity to convey nonlexical linguistic and high-lever cues. Among these features, pitch reveals the frequency at which the vocal folds vibrate and relies greatly on broad classes of sounds [29]. Pitch is investigated in this paper to indicate the characters of different emotion types.

**Figure 1:**
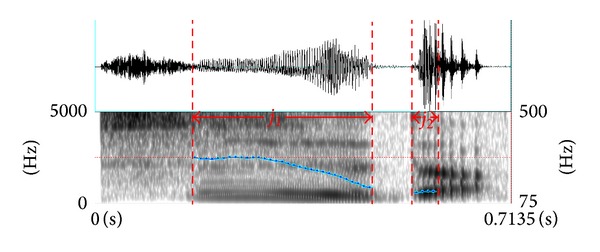
Example of segment boundaries estimation for the phrase “shi de.” The vertical bars represent the segment boundaries from the critical points of pitch contours.

The production of speech is the process of setting the air in rapid vibration. Periodic air pulses passing through vibrating vocal chords make voiced sounds, for example, “a” and “i” while unvoiced sounds such as “s” and “sh” are created by forcing air through a constriction in vocal tract, producing turbulence that is more noise-like. In this case, the pitch of an utterance is discontinuing. We can easily divide speech into voiced and unvoiced regions by detecting the points where the pitch contour appears or disappears.  Figure 1 shows the waveform, spectrum, and pitch of phrase “shi de.” The voiced segment alternates with the unvoiced one. The boundaries of voiced and unvoiced speech are represented by vertical dotted bars. The pitch contour of the voiced speech is defined as pitch envelope here. The statistics and analysis of pitch parameters take the pitch envelope as a unit, as it could indicate the average level of the speaker's voice frequency, which varies greatly under different emotional states.

**Definition 1**

Let *J* = {*j*_1_, *j*_2_,…, *j*_*T*_} be a pitch sequence of an utterance and let *T* be the frame numbers. *j*_*i*_ = 0 for the pitch of unvoiced segments and *j*_*i*_ > 0 for the pitch of voiced segments. The pitch envelope is denoted by J* = {*j*_*i*_ | *i* = *n*, *n* + 1,…, *m*} and satisfies the following constraint:

1. *j*_*i*_ ≠ 0,
2. *j*_*n*−1_ = 0, *j*_*m*+1_ = 0,
3. 0 ≤ *n* ≤ *i* ≤ *m* ≤ *T*.

The mean value of pitch envelope (PEM) can be calculated as

(1) $$\begin{array}{c} {\overset{⃑}{J}}^{*}=\frac{1}{n-m+1}\overset{n}{\underset{i=m}{\sum }}j_{i}, \end{array}$$

where *m* and *n* are the numbers of the start and the end frame of a pitch envelope.

**Figure 2:**
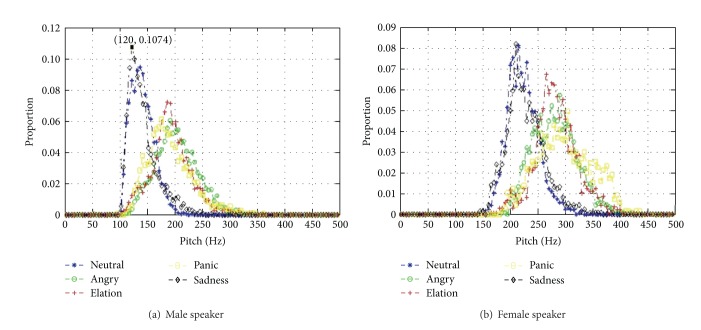
The probability distribution of PEM for the male (a) and female speakers (b) under the five emotion states.

The pitch of a man's voice falls under low pitch frequency (60–250 Hz), whereas woman's voice is of the high pitch type (120–500 Hz). The distributions of PEM for male and female are studied separately.  Figure 2 shows the probability distribution of PEM for all the sentences in MASC under the five emotion states. In particular, Figure 2(a) is the probability distribution of PEM with male's speech, while Figure 2(a) is the probability distribution of PEM with female's speech. The whole pitch frequency from 0 Hz to 500 Hz is equally divided into 100 scales with 5 Hz width each. For example, the point “100 Hz” on the abscissa represents the scales from 95 Hz to 100 Hz. The value on the ordinate corresponding to 100 Hz represents the proportion of voiced speech whose PEM falls in (95, 100) for each emotion. The point (120, 0.1074) means that there is 10.74% of PEM that falls in (115, 120) scope.  Figure 2 demonstrates the probability distribution of PEM over 5 emotion types that can be divided into two groups. The neutral and sadness speech have similar distribution with smaller mean and variance value. The PEM probability distribution of anger, elation, and panic has larger mean and variance value. In this case, we assume that the voiced speech that has high PEM value is heavily affected by speaker's emotional mood. We partition the voiced speech into two groups according to a threshold pitch value: (1) the class that is highly different from neutral speech mainly includes the pitch envelop of anger, elation, and panic; (2) the class that is slightly different from neutral speech, mainly includes the pitch envelop of neutral and sadness. Both the male and female's speech have similar distribution, except that all the PEM of female are much higher than that of males'.

**Figure 3:**
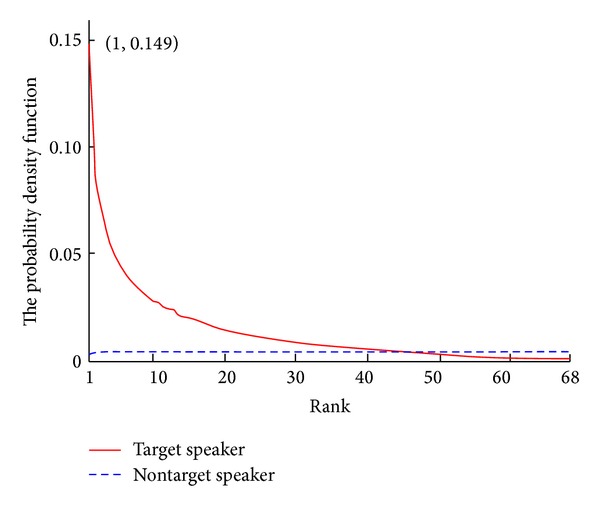
The frame-level score rank's probability density functions for target speakers and nontarget speakers over 68 subjects in MASC.

Thus, we can draw two kinds of important information. First, the PEM selection parameters should be set differently for male and female speakers as their dissimilarity distribution. Second, not all frames of the utterance are impacted dramatically by affective speech. In the speaker recognition task, the majority of frames of the test speech give the maximum probability (rank 1 score) to the target speaker (TS). In this case, the utterance could be correctly classified. However, the target speaker could not get the rank 1 score from all the test frames. In particular when the mood state of speakers shifts from neutral to affective, the vocal and prosody features are also changed. Some test frames give their confidence to a nontarget speaker (NTS) mistakenly because of the mismatch of emotions between the speaker models and the test utterances. With the number of frames that assign the maximum probability to the NTS becoming enormous, the score of the NTS could be comparable or even higher than that of the TS.  Figure 3 shows the frame-level score rank's probability density functions for target speakers and nontarget speakers. The number on the abscissa represents the rank of score for each frame. For instance, the point (1, 0.149) in the red curve of target speaker means 14.9% of frames give the rank 1 score to the target speaker.

The reason why the test utterance is misclassified is not due to a nontarget speaker doing well but rather to a true speaker's model doing poorly. It is assumed that most frames still give the maximum likelihood to the target model, as they are not easily changed with the slight expressive corruption, while part of the frames that have large variations in *F*_0_ may be affected by the emotion state change of speakers, and we define these frames as bad frames in this paper. To overcome the mistaken decisions induced by bad frames, we strengthen the roles of the good frames by giving them weight to exhibit their importance based on cost-sensitive learning.

Combined with the analysis of pitch, we divided the frames into two parts according to the variation of the *F*_0_ value. The voiced part with high PEM that is heavily affected by the expressive speech (HA) is taken as bad frames. On the contrary, the voiced part that is slightly affected (SA) together with the unvoiced is considered as good frames.

## 4. Cost-Sensitive Speaker Recognition

### 4.1. Definition of Cost-Sensitive Learning

Cost-sensitive classification is based on a set of weights defining the expected cost when an object is misclassified. First, we give the definition for cost-sensitive classification problem.

Let *x* ∈ *R* ^*n*^ be a feature vector, let {1, 2,…, *N*} be the label set of *N* classes, and let *C* be a *N*∗*N* cost matrix with entries *c*_*i*,*j*_. *c*_*i*,*j*_ are the cost of misclassifying an example of class *i* to class *j*; both *i* and *j* belong to {1, 2,…, *N*}. *c*_*i*,*j*_ > 0 if *i* ≠ *j* and *c*_*i*,*j*_ = 0 if *i* = *j*:

(2) $$\begin{array}{c} C=\left[\begin{array}{cccc} 0 & c_{1,2} & \ldots & c_{1,N}\\ c_{2,1} & 0 & \ldots & c_{2,N}\\ \ldots & c_{i,j} & 0 & \ldots \\ c_{N,1} & \ldots & c_{N,N-1} & 0 \end{array}\right]. \end{array}$$

Here, *c*_*i*,*i*_ = 0 is the correct classification. The expectation cost of class *i* can be computed by

(3) $$\begin{array}{c} c_{j}=\overset{N}{\underset{i=1}{\sum }}c_{i,j}. \end{array}$$

The cost-sensitive learning can be defined as follows.

**Definition 2**

Let *P*(*X* | *Y*) be the unknown joint distribution of *X* and *Y*. Let *F* be a set of mappings from *X* to *Y*. The cost-sensitive learning procedure is to select a mapping *f* (*f* ∈ *F*), to minimize the risk functional *R*(*f*), defined as

(4) $$\begin{array}{c} R\left(f\right)=E_{P(X\;|\;Y)}c_{y,f(x)}=\int \left[\overset{N}{\underset{y=1}{\sum }}c_{y,f(x)}P\left(y\;|\;X\right)\right]p\left(x\right)dx. \end{array}$$

It is easy to recognize that when given *c*_*i*,*j*_ = 1 if *i* ≠ *j*, (4) reduces to the standard classification.

### 4.2. Speaker Recognition Task

In the speaker recognition task, given a group of *N* known speakers model Λ = {*λ*_1_, *λ*_2_,…, *λ*_*N*_} and a sample of test speech with *T* frames, the likelihood of *X* that belongs to the *i*th speaker can be written as *P*(*X* | *λ*_*i*_) according to Bayes' rule. In the case that frames in the utterance are independent, *P*(*X* | *λ*_*i*_)  could be expressed as

(5) $$\begin{array}{c} P\left(X\;|\;\lambda_{i}\right)=\overset{T}{\underset{t=1}{\prod }}p\left(x_{t}\;|\;\lambda_{i}\right). \end{array}$$

Obviously, there are relations between frames and frames, and the *P*(*X* | *λ*_*i*_) can be rewritten as

(6) $$\begin{array}{c} P\left(X\;|\;\lambda_{i}\right)=\overset{T}{\underset{t=1}{\prod }}f\left(p\left(x_{t}\;|\;\lambda_{i}\right)\right). \end{array}$$

Equation (5) is a special case of (6) when *f*(*x*) = *x*.

In the process of computing the test utterance on the speaker model, the score is always mapped to the log domain for calculation facilitation as follows:

(7) score(X ∣ λi)=log⁡p(X ∣ λi)=log⁡∏t=1Tf(p(xt ∣ λi))=∑t=1Tf(log⁡p(xt ∣ λi)).

According to the frame selection conducted in Section 3, the frames in the SA part and unvoiced part are reweighed to strengthen their confidence to the maximum likelihood model. Thus, for a special speaker *i*, the utterance level score of a *T* frame speech sequence is defined as

(8) $$\begin{array}{c} \text{score}\left(X\;|\;\lambda_{i}\right)=\overset{T_{1}}{\underset{t=1}{\sum }}f\left(\log p\left(x_{t}\;|\;\lambda_{i}\right)\right)+\overset{T_{2}}{\underset{t=1}{\sum }}\log p\left(x_{t}\;|\;\lambda_{i}\right), \end{array}$$

where *T*_1_ is the number of frames in SA and unvoiced part, *T*_2_ is the number of frames in HA part, respectively, and *T* = *T*_1_ + *T*_2_.

Given the frame vector *x*_*t*_ in the SA part and the speaker *i*, the cost-sensitive function can be assumed as

(9) $$\begin{array}{c} f\left(\log p\left(x_{t}\;|\;\lambda_{i}\right)\right)=\overset{N}{\underset{y=1}{\sum }}c_{y,i}*\log p\left(x_{t}\;|\;\lambda_{i}\right). \end{array}$$

Note that cost matrix *C* only needs to be computed once when function *L* is defined.

It is obvious that the reweight function should meet the rule that the frame score and the cost matrix *L* are in direct ratio.

### 4.3. Cost-Sensitive Parameters Learning

To deal with the class-dependent cost-sensitive learning problem, the data space expansion technique is adapted [30, 31]. Each example is expanded to *N* examples of different classes. The weights of *N* examples are decided based on the loss of the corresponding misclassifications. When a test utterance is compared with a certain speaker model, the sum loss of its expanded *N* examples is in proportion to the loss of classifying it to that speaker model. The details of the expansion technique are given as follows.

Assume that in a speaker classification task, sample *X* is assigned to speaker model *Y* by classifier *f*(*X*). *C*_*y*_ is positive as well as being not less than the largest misclassification loss in order to keep weights of expanded examples positive. The loss of *f*(*X*) on the example (*X*, *Y*) is defined as

(10) cy,f(x)=∑i=1Ncy,i−∑i=1Ncy,iI(f(x)≠i)=∑i=1Ncy,i−∑i=1Ncy,iI(f(x)≠i)+(K−1)cy−(K−1)cy=∑i=1N(cy−cy,i)I(f(x)≠i)−∑i∈{1,…,N}∖yN(cy−cy,i),

where *I*(*x*) is a step function with value 1 if the condition in the parenthesis is true and 0 otherwise.

The expanded examples (*X* ^*n*^, *Y* ^*n*^) with weights *w*_*y*,*n*_ are defined as

(11) $$\begin{array}{c} X^{n}=X,\;\;Y^{n}=n,\;\;w_{y,n}=\left(c_{y}-c_{y,n}\right). \end{array}$$

Substituting (11) into (10), we can get

(12) $$\begin{array}{c} c_{y,f(x)}=\overset{N}{\underset{i=1}{\sum }}w_{y,i}I\left(f\left(x\right)\neq i\right)-\varphi \left(y\right), \end{array}$$

where *φ*(*y*) = ∑_*i*∈{1,…,*N*}∖*y*_^*N*^ *w*_*y*,*i*_.

The loss of *f*(*x*) on the example (*X*, *Y*) could be computed by a weighted loss of *f*(*x*) on expanded examples minus a variable irrelevant to *f*(*x*). The weights can modify the distribution on (*X*, *Y*) and produce a new one as well. In other words, cost-sensitive learning can be reduced to the standard classification [30].

## 5. System Architecture

Our previous work presented a pitch envelope based frame-level score reweighted speaker recognition framework [7]. The main contribution of this work is to introduce the cost-sensitive learning to reweigh the score. The testing process of the proposed speaker recognition system relies on 3 modules: gender identification, PEM based pitch envelop selection, and frame-level probability reweighed.

The purpose of the gender identification is to set different PEM threshold for male speakers and female speakers. This process is taken before frame selection. Given an utterance, the Mel frequency cepstral coefficients (MFCC) feature is extracted and tested with both male and female GMM models to produce the likelihood scores. The utterance is classified to the gender that has higher likelihood score. Corresponding frame selection thresholds are set and adopted based on the result of gender identification.

In the process of the PEM based pitch envelop selection, the variation of pitch distribution under different emotional states is analyzed and compared with PEM threshold. The voiced envelop frames whose mean pitch value is smaller than threshold and the unvoiced part are chosen for reweighting.

The score reweight step aims to strengthen the confidence of the selected speech segments and optimize the final accumulated frame scores over the whole test utterance.

## 6. Experiment and Discussion

### 6.1. Experiment Settings

The evaluation task conducted in the experiments is text-independent and closed-set speaker recognition. The front end processing of speech single is as follows. A 13-dimensional Mel frequency cepstral coefficients (MFCC) vector is extracted from the preemphasized speech signal every 16 ms using a 32 ms Hamming window. A simulated triangular filter bank on the DFT spectrum is used to compute the Mel cepstral vector. Delta-cepstral coefficients are then computed and appended to the 13-dimensional cepstral vector, producing a 26-dimensional feature vector. The speaker classification, the GMM, consists of 32 mixture components. In the gender identification, two 1024 mixture GMMs, male and female model, are built with MAP method using the speech from the development subset. The statistical *F*_0_ thresholds of PEM for the female and male speakers are set as 289 Hz and 189 Hz, respectively.

### 6.2. The Baseline System with Neutral Models

**Figure 4:**
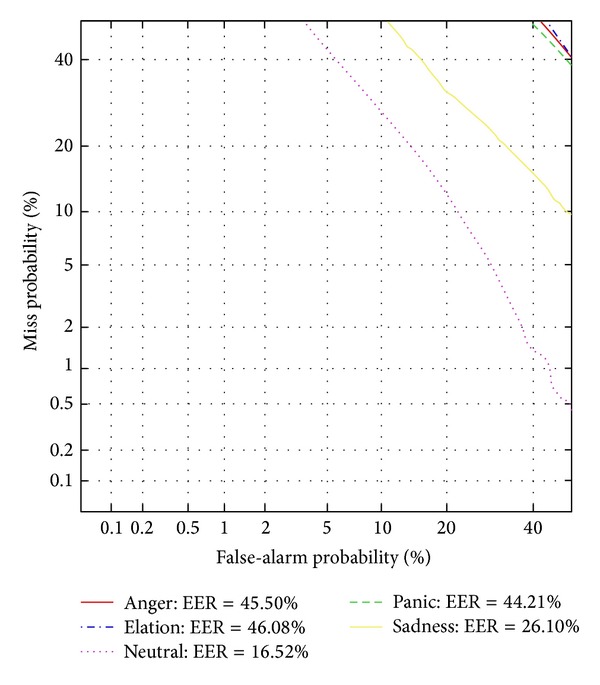
DET curves for the traditional speaker models trained with neutral speech only.

The aim of the first experiment is to capture the fluctuation in the system performance with various affective speeches. The speaker models are built with paragraph part on neutral speech, and the test utterances are in anger, elation, panic, sadness, and neutral, respectively.  Figure 4 gives the verification results with the five types of affective speech tested independently with neutral speaker models. The verification performance will decline greatly when the system is involved with affective utterances for testing. It is clear that the consistence affective state of the training and testing utterances is important. The verification performance for speech in anger, elation, and panic drops more sharply than that in sadness. It is reported that the speakers would have a much higher arousal level mood when they are in the emotion of anger, elation, and panic than that of sadness [32]. This is one of the possible reasons that the EER of test speech in sadness state goes down to 26.1%, while the EER of test speech in other three affective states have a sharper drop.

### 6.3. Experiment Results and Discussions

The identification rates (IR) of the standard accumulated approach and the CSSR on emotional speech of anger, elation, neutral, panic, and sadness are shown in Table 1. The enhancement of IR for speech in anger, elation, and panic achieves 11.94%, 13.53%, and 9.84%, respectively, which is significantly greater than that achieved for speech in sadness and neutral. A possible reason is that when speakers are in the emotion of anger, elation, and panic, they would have a much higher arousal level mood which makes more speech envelops with high PEM. In other words, the speech in anger, elation, and panic has a much greater number of the bad frames. Once the confidence of good frames are strengthened for the speech, the identification rates of speaker recognition are easily promoted.

**Figure 5:**
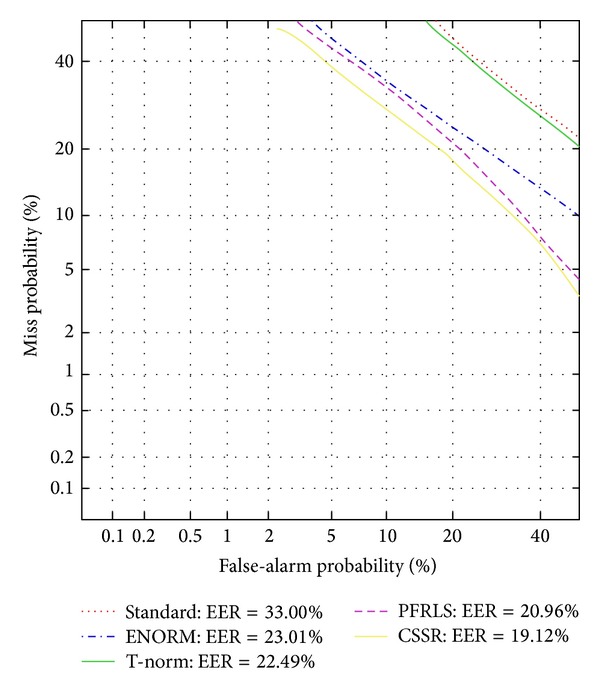
DET curves for the baseline, T-norm, ENORM, PFLSR, and CSSR based speaker verification system.

We also apply the proposed method to speaker verification task and compare it with other score normalization methods, as shown in Figure 5. The performance measured by the detection error tradeoff function (DET) as well as equal error rate (EER). The EER is calculated as the operating point on the DET curve where the false-alarm and missed-detection rates are equal. Figure 5 shows the promise of the new approach. Evaluation results clearly show that CSSR technique outperforms the standard accumulated approach, T-norm, and ENORM methods for speaker verification on affective speech.

## 7. Conclusion and Discussion

In this paper, we introduce cost-sensitive learning to speaker recognition system with emotional speech. Based on an emotion robustness framework, cost-sensitive parameters are used to refine the probability of the slightly affected envelops and to strengthen the confidence of the target speakers. Promising results are achieved in both speaker identification and speaker verification system. In future work, more effective algorithms of the frame selection and clustering recognition method [33] may be suggested to be employed in this system. On the other hand, the emotional parameters associated with specific speakers can also be considered as the characteristics in the recognition of their speeches [34].

## Figures and Tables

**Table 1:** Comparison of system performance under different types of affective speech (%).

Method	Baseline	CSSR
Anger	21.80	33.74
Elation	22.70	36.23
Neutral	94.40	95.63
Panic	26.30	36.14
Sadness	51.13	54.67
Total	**43.27**	**51.28**
